# Effect of *Astragali radix* extract on pharmacokinetic behavior of dapagliflozin in healthy and type 2 diabetic rats

**DOI:** 10.3389/fphar.2023.1214658

**Published:** 2023-10-10

**Authors:** Wandi Du, Jiarong Hu, Jingru Liang, Xiaolei Yang, Boyu Fang, Guo Ma

**Affiliations:** Department of Clinical Pharmacy, School of Pharmacy, Fudan University, Shanghai, China

**Keywords:** dapagliflozin, *Astragali radix*, *Astragali radix* extract, type 2 diabetes mellitus, pharmacokinetics, UPLC-MS/MS

## Abstract

**Objective:** This study aimed to investigate effect of antidiabetic herb *Astragali Radix* (AR) on pharmacokinetic behavior of dapagliflozin (DAPA) in healthy rats and type 2 diabetes mellitus (T2DM) rats.

**Methods:** The T2DM rats were induced by high-fat diet (HFD) and intraperitoneal injection of streptozotocin (STZ). Concentrations of DAPA in healthy and T2DM rat plasma were determined by UPLC-MS/MS method. Effect of AR extract (ARE) on pharmacokinetic behavior of DAPA in healthy and T2DM rats was evaluated, respectively.

**Results:** The diabetes status and co-administrated with ARE significantly affected pharmacokinetic behaviors of DAPA in the rats. Compared to that in healthy rats, *t*_max_ of DAPA significantly shortened, its *C*_max_ significantly increased in T2DM rats, and its *t*_1/2_, *V*, AUC, CL and MRT kept unchanged. When ARE was co-administrated with DAPA, *C*_max_ of DAPA significantly increased, its *t*_max_ and MRT significantly decreased, and its *t*_1/2_, *V*, AUC and CL kept unchanged in healthy rats. *t*_max_ and *C*_max_ of DAPA significantly decreased, its *t*_1/2_ and *V* significantly increased, and its AUC, CL and MRT were unchanged in T2DM rats when ARE was co-administrated with DAPA. Co-administration of DAPA and ARE promoted absorptive rate of DAPA, increased its extravascular tissue distribution, and prolonged its duration of action. ARE did not cause accumulation of DAPA *in vivo*.

**Conclusion:** Both disease status of T2DM and co-administration of ARE affect pharmacokinetic behavior of DAPA *in vivo*. Potential pharmacokinetic interactions may occur *in vivo* when herbs and drugs are co-administrated, which may affect efficacy and safety of drugs.

## 1 Introduction

Diabetes mellitus (DM) is a chronic systemic metabolic disorder caused by combination of genetic and environmental factors over an extended period of time (Cole and Florez, 2020). Type 1 diabetes mellitus (T1DM) and type 2 diabetes mellitus (T2DM) are two major forms of DM, primarily triggered by pancreatic beta cell dysfunction, insulin secretion deficiency and insulin resistance (Eizirik et al., 2020). At least 90% of all cases of DM are T2DM (American Diabetes Association Professional Practice Committee, 2022). The 10th edition IDF Diabetes Atlas reported that, global diabetes prevalence in 20–79 years old was estimated to be 10.5% (536.6 million people) in 2021, and will rise to 12.2% (783.2 million) in 2045 in the world (Sun et al., 2022). The patients with DM were estimated to be 140 million in 2021, 174 million in 2045 in China (Sun et al., 2022). Long period exposure of patients to high blood glucose affects the function of multiple tissues and organs, leading to a range of complications such as microvascular damage (e.g., retinopathy, nephropathy, neuropathy) and macrovascular events (e.g., atherosclerotic ischaemic) (Schmidt, 2018; Cosentino et al., 2020). The epidemic of DM and its complications poses a major threat to global health.

Antidiabetic drug treatment, lifestyle intervention as well as education and management of DM are the main measures for prevention and treatment of DM (Zheng et al., 2018). Popular antidiabetic drugs include insulin, biguanides, sulfonylureas, glinides, dipeptidyl-peptidase 4 (DPP-4) inhibitors, glucagon-like peptide-1 (GLP-1) receptor agonists and sodium-dependent glucose transporters 2 (SGLT-2) inhibitors (Cosentino et al., 2020). However, despite a variety of different treatment options, the above antidiabetic drugs have their limitations, e.g., single anti-diabetes target, leading to hypoglycemia, gastrointestinal reactions, weight gain, genitourinary tract infection, and so on. When DM and its complications are not well treated with single antidiabetic drugs, two or more antidiabetic drugs or herbs are usually co-administrated to improve efficacy and reduce incidence of adverse events.

Dapagliflozin (DAPA) is a highly selective SGLT2 inhibitor, and is used to treat T2DM by reducing renal glucose reabsorption and promoting urinary glucose excretion (Kasichayanula et al., 2014). As the first approved SGLT2 inhibitor in treating T2DM in the world, it is widely used in clinical practice at present. Clinically meaningful improvements in measurements of glycemic control have been demonstrated in patients with T2DM when treated with DAPA as monotherapy, or in combination with other antidiabetic drugs. The fixed-dose combination of DAPA with other antidiabetic drugs, e.g., DAPA in combination with Metformin (Xigduo) (Cheng et al., 2021), Saxagliptin (Qtern) (Garnock-Jones, 2017), or insulin (Forxiga) (Dhillon, 2019), exerts synergistic antidiabetic effects based on complementary mechanisms of action. However, co-administration of DAPA and other antidiabetic drugs increases risk of adverse events, e.g., hypoglycemia, urethral and reproductive tract infections. Co-administration of antidiabetic herbs (e.g., *Astragali Radix*) and antidiabetic drugs (e.g., DAPA) can improve antidiabetic effect and reduce risk of adverse events since herbs usually display favorable antidiabetic effect and relatively high safety (Gupta et al., 2017).

*Astragali Radix* (AR), also known as Huangqi in China, is one of the most widely used herbal medicines. It is the dried root of *Astragalus* membranaceus (Fisch.) Bge. or *Astragalus* membranaceus (Fisch.) Bge. var. mongholicus (Bge.) Hsiao (Fu et al., 2014). More than 200 compounds have been identified in AR, including flavonoids (e.g., calycosin, calycosin-7-β-glucoside, formononetin, ononin), saponins (e.g., astragaloside Ⅰ-Ⅶ) and polysaccharides (e.g., astragalus polysaccharide) (Zhang et al., 2021). AR, its extracts (i.e., ARE) and their ingredients exhibit a wide variety of pharmacological activities, e.g., antidiabetic, antioxidant, antitumor, antiviral, anticonvulsive, immunomodulatory, liver and kidney protection, diuretic and expectorant (Zhang et al., 2020; Sheik et al., 2021), and are usually used to treat T2DM, diabetic kidney disease (Li et al., 2011; Chan et al., 2021; Tang and Zhang, 2022; Gao et al., 2023), cancer (Li et al., 2023), myocardial fibrosis (Ren et al., 2023), multiple sclerosis (Peng et al., 2023), interstitial lung disease (Zhou et al., 2023) and neurodegenerative disorders (Abd Elrahim Abd Elkader et al., 2022) in clinical and folk practice for a long history in Asia. Due to its favorable efficacy and safety in the treatment of T2DM and its complication, AR and AR-containing formulas are often co-administered with DAPA to enhance their antidiabetic effects, reduce their dosage and adverse events.

Pharmacokinetic properties of DAPA and its co-administration with other antidiabetic drugs (e.g., metformin, pioglitazone, sitagliptin, glimepiride, voglibose) have been comprehensively studied (Kasichayanula et al., 2014). However, up to now, pharmacokinetic interactions between DAPA and herbs such as AR, have not been studied. Effect of ARE on pharmacokinetic behavior of DAPA in healthy and T2DM rats was explored in this study, which will provide an important reference and beneficial guidance for treatment of DM by co-administration of antidiabetic herbs and drugs.

## 2 Materials and methods

### 2.1 Materials and reagents

Astragali Radix roots were provided by Beijing Tong-Ren-Tang Pharmaceutical Co., Ltd. (Tianjin, China). Dapagliflozin (purity ≥ 98%) and empagliflozin (purity ≥ 99.5%) were provided by TargetMol (Shanghai, China). Streptozotocin (STZ), formic acid (HPLC grade), methyl tert-butyl ether (MTBE, HPLC grade), citric acid (analytical grade), sodium citrate (analytical grade), sodium carboxymethyl cellulose (CMC-Na), were provided by Aladdin Bio-Chem (Shanghai, China). Methanol and acetonitrile (both HPLC grade) were provided by Xingke High Purity Solvents (Shanghai, China). Ammonium formate (LC-MS grade) was provided by Jingming Biotechnology (Beijing, China). 45% of high fat feed (XTHF45-1: 22.5% crude protein, 24.2% crude fat, 3.2% crude fiber, 5.6% crude ash, 1.2% calcium, 0.8% total phosphorus) was provided by Xie Tong Organism (Jiangsu, China).

### 2.2 Solution preparation

The AR roots were ground into fine powder, and 10 times volume of water was added to reflux extraction. Each extraction time was 120 min, and the extraction was performed three times. The extracts were combined and concentrated to obtain ARE solution. The ARE solution was lyophilized under reduced pressure to obtain ARE freeze-dried power (30% of ARE content in the raw medicinal materials). The DAPA solution (0.25 mg/mL) was prepared by dissolving DAPA in 0.5% CMC-Na solution. STZ solution (1%) was prepared by dissolving STZ in 0.1 mol/L ice-cold citrate buffer (pH 4.5).

### 2.3 Instrumentation and chromatographic conditions

An AB SCIEX (USA) Exion LC system consisting of vacuum degasser, binary pump and auto-sampler were used for solvent and sampler delivery. Chromatographic separation was achieved using Agilent Poroshell 120 EC-C_18_ column (2.1 × 100 mm, 1.9 μm). The column temperature was maintained at 40°C. The mobile phase consisted of 0.1% formic acid-1mM ammonium formate-water solution (A) and acetonitrile (B) was used, and the flow rate was set at 0.3 mL/min. The gradient elution program was as follows: 0–2.0 min (90%–65% A), 2.0–6.0 min (65%–60% A), 6.0–7.0 min (60%–90% A), 7–10 min (90% A). The processed samples were stored at 4°C in an auto sampler. The sample injection volume was 2 μL. An AB SCIEX (USA) Q-TRAP 6500 model mass spectrometer (MS) equipped with a turbolon spray ionization (ESI) source was used for mass analysis and detection. DAPA and the internal standard (IS, i.e., empagliflozin) were analyzed in positive ion mode with multiple reaction monitoring (MRM) pairs: m/z 426.1–355.0 for DAPA and m/z 468.1–355.1 for IS. The ion spray voltage was set at 5500V. The curtain gas, ion source gas 1 and ion source gas 2 were set at 35, 60 and 50 psi, respectively. The source temperature was kept at 200°C. The collision gas was set at low flow rate. The declustering potential and collision energy were 35 V and 19 V for DAPA, 40 V and 21 V for IS.

### 2.4 Preparation of stock solution and working solution

DAPA was dissolved in 50% methanol to make DAPA stock solution with final concentration of 1 mg/mL. DAPA calibration working solutions were prepared by dilution of the DAPA stock solution with 50% methanol. Final concentrations of DAPA were 76.9, 153.8, 307.5, 615.0, 1230.0, 2460.0, 4920.0, 9840.0 ng/mL, respectively. Empagliflozin (IS) solution (427.5 ng/mL) was prepared and diluted with 50% methanol. Stock solution and working solutions of DAPA were stored at -20°C and 4°C, respectively.

### 2.5 Preparation of calibration standards and quality control (QC) samples

Calibration standard samples were obtained by spiking 5 μL of DAPA working solution and 5 μL IS solution with 50 μL of blank rat plasma. Then, 500 μL of MTBE was added into the calibration standard samples for liquid-liquid extraction. The extracted sample was vortexed for 3 min and centrifuged 5 min (4000 rpm, 4°C) to separate the organic and aqueous phases. The upper organic phase was transferred and evaporated by nitrogen blowing, then 100 μL of 50% methanol was added to re-dissolve the samples. The final concentrations of DAPA in the simulative rat plasma were 7.7, 15.4, 30.8, 61.5, 123.0, 246.0, 492.0, 984.0 ng/mL, respectively. The QC samples were processed in the same method with the final concentration of 7.62 (lower limit of quantification, LLOQ), 33.5 (low QC, LQC), 244 (medium QC, MQC), 975 (high QC, HQC) ng·mL^−1^ for DAPA. All these solutions were stored at 4°C.

### 2.6 Plasma samples preparation

The rat plasma samples (50 μL), IS working solution (5 μL), 50% methanol (5 μL), and MTBE (500 μL) were mixed, then were vortexed for 3 min and centrifuged for 5 min (4000 rpm, 4°C). The organic phase was transferred to a new centrifuged tube and evaporated to dryness with a stream of nitrogen at room temperature. The dried samples were redissolved with 100 µL of 50% methanol and vortexed for 1 min, then 2 µL of supernatant fluid was injected into the LC-MS/MS instrument for analysis.

### 2.7 Method validation

The LC-MS/MS method was validated according to the guidelines of the Bioanalytical Method Validation Guidance for Industry for the US Food and Drug Administration (US-FDA, 2018) and Chinese Pharmacopoeia Commission (2020). Selectivity, calibration curve, limit of detection (LOD), LLOQ, precision, accuracy, matrix effects, recovery, and stability of the analysis method under various conditions were validated.

#### 2.7.1 Selectivity

Selectivity of the analysis method was examined by comparing the chromatograms of blank plasma samples obtained from six rats, blank plasma spiked with the analytes (at LLOQ) and IS, and plasma samples of the rats administered DAPA and ARE. In the absence of interference, the peak area of the analyte in the blank plasma should be less than 20% of the LLOQ and 5% of the IS within the retention time.

#### 2.7.2 Calibration curve, LOD and LLOQ

The calibration standard samples (*n* = 8) were assayed for 3 days. The linearity for each analyte was constructed by the weighted (1/x^2^) least square linear regression of the peak area ratios of the analytes to the corresponding IS against concentrations. LOD was calculated as the final concentration of DAPA and IS producing a signal-to-noise ratio of 3. LLOQ was considered as the lowest concentration of the calibration curve. Accuracy of the back-calculated concentrations of the calibration standards should be within ± 15% of the nominal value, except for LLOQ should be within ± 20%.

#### 2.7.3 Precision and accuracy

Precision and accuracy of the analysis method were evaluated by analyzing QC samples prepared at four concentration levels (low, medium, and high concentration) and LLOQ on three separate days. Precision was calculated as the relative standard deviation (*RSD*, %), and accuracy was expressed as the relative error (*RE*, %). RE of the mean concentration at each QC level will be within ± 15% of the nominal values, except for the QC samples at LLOQ which will be within ± 20% of the nominal values. *RSD* of measurements at each QC level will be within 15% except for the QC samples at LLOQ which will be within 20%.

#### 2.7.4 Matrix effect and extraction recovery

Matrix effect for the analyte was determined by comparing peak area of the analyte in blank plasma samples (three replicates of LQC and HQC samples, respectively) from six different rats with peak area of the analyte in pure solution (50% methanol). Extraction recovery of the analyte was assessed by comparing peak area of the analyte in the plasma pre-extracted with MTBE from six different rats (three replicates of samples with DAPA at low and high concentration, respectively) to peak area of the analyte in the LQC and HQC samples (three replicates) at the same level, respectively.

#### 2.7.5 Stability

Stability of DAPA in rat plasma was evaluated by analyzing six replicates of QC samples at low and high concentrations under different conditions. Short-term stability was investigated by keeping the samples at room temperature (25°C) for 12 h and autosampler (4°C) for 24 h, respectively. Freeze-thaw stability was investigated by three freeze-thawing cycles, i.e., the samples were frozen for 7 d at -80°C and thawed 3 h at room temperature for each cycle. Long-term stability was investigated by keeping the samples at -80°C for 1 month.

### 2.8 Animal experiments

Male Sprague Dawley (SD) rats (180–220g, 6–8 weeks old) were provided by Animal Experimentation Centre, School of Pharmacy, Fudan University (Shanghai, China). All the experimental procedures for this study were reviewed and approved by Experimental Animal Ethics Committee of School of Pharmacy, Fudan University, which was in strict compliance with the International Code for the Care and Use of Experimental Animals (approval number:2020-04-LY-MG-01). All the animals were housed under standard laboratory conditions of temperature (25°C ± 2°C), constant humidity (55 ± 5%), and light (12 h dark/light cycle), with freely available food and purified water. After 1 week of acclimation, 12 rats were randomly selected as the healthy group that was fed with the ordinary feed. The remaining 20 rats were modeled for T2DM that were fed with high-fat diet (HFD). After 4 weeks, the modeled rats fed HFD were fasted overnight, and measured fasting blood glucose (FBG), then intraperitoneally injected with STZ (35 mg/kg, inject once). One week later, the FBG was measured again, and only those rats whose FBG were over 16.7 mmol/L were considered to be T2DM rats. The rats whose FBG did not meet the criterion were not included in the following experiment.

Dose of 1 mg/kg for DAPA used in the study was recommended based on the package insert of DAPA and dose conversion per unit weight from humans to rats (6.3 times). ARE was administered to the rats with the dose of 300 mg/kg, which was selected by converting the recommended doses of AR for adults in practice to the doses of ARE for the rats, according to the Pharmacopoeia of the People’s Republic of China. The healthy rats and T2DM rats were randomly assigned to two groups (*n* = 6 each group). The CD group was the healthy rats intragastrically administered with DAPA in a single dose (1 mg/kg). The CDA group was the healthy rats intragastrically administered with ARE (300 mg/kg) once a day for seven consecutive days, and intragastrically administered with DAPA (1 mg/kg) at the same time on the seventh day. The MD group was the T2DM rats intragastrically administered with DAPA in a single dose (1 mg/kg). The MDA group was the T2DM rats intragastrically administered with ARE (300 mg/kg) once a day for seven consecutive days, and intragastrically administered with DAPA (1 mg/kg) at the same time on the seventh day.

The rats were fasted for 12 h prior to the pharmacokinetic experiments, and they were allowed to drink freely. The rat blood was collected from retro-orbital sinus pre-dosing at 0, 0.25, 0.5, 1, 2, 4, 6, 8, 12, 24 h. The collected blood was added into the centrifuge tube with EDTA-2Na, shaken well, and centrifuged for 15 min (3000 rpm, 4°C), and then stored at -80°C until further analysis. The samples were processed by the procedure described above.

### 2.9 Statistics

MaS Studio PK Software 1.2.0 (Mathematical Pharmacology Professional Committee of China, Shanghai, China) was used to calculate pharmacokinetic parameters with non-compartmental analysis. The pharmacokinetic parameters included *t*_1/2_ (time required to eliminate half of plasma drug concentration), *t*_max_ (time to maximum plasma concentration), *C*_max_ (maximum plasma concentration), AUC_(0-t)_ (area under the concentration-time curve from 0 to *t* time), AUC_(0-inf)_ (area under the concentration-time curve from 0 to infinite time), *V* (apparent volume of distribution), CL (clearance of drug plasma volume per time unit), and MRT (mean retention time). GraphPad Prism 9.0 (La Jolla, CA) were used to statistically analyze the above-mentioned pharmacokinetics parameters. Statistical comparisons of pharmacokinetic parameters of DAPA between the healthy rats and T2DM rats, and DAPA used alone and combination of ARE group were conducted using one-way ANOVA. *p* < 0.05 was considered as statistically significant.

## 3 Results

### 3.1 T2DM rat validation

FBG of 20 rats was 5.33 ± 1.26 mmol/L after 4 weeks of HFD and before STZ injection. After 1 week of STZ injection, 16 rats met the criteria of T2DM (i.e., FBG ≥ 16.7 mmol/L), and their mean FBG was 19.78 ± 2.11 mmol/L with a moulding rate of 80%. 4 rats were not included in the following experiment because their FBG did not meet the criterion of T2DM rats.

### 3.2 Method validation

#### 3.2.1 Selectivity

**FIGURE 1:**
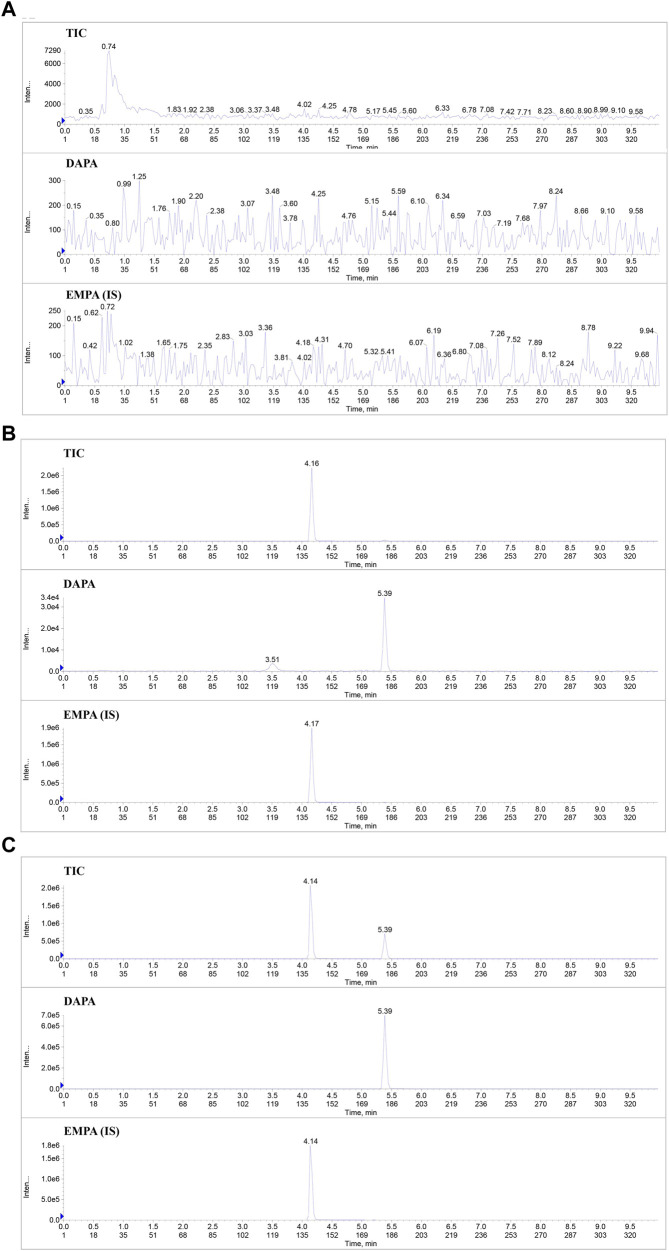
Typical chromatograms of DAPA and IS in the rat plasma samples. **(A)**, Total ion flow chromatogram (TIC) and the extracted ion flow chromatogram of DAPA and IS in the blank rat plasma. **(B)**, TIC and the extracted ion flow chromatogram of DAPA and IS in the blank rat plasma spiked with the analytes (at LLOQ) and IS. **(C)**, TIC and the extracted ion flow chromatogram of DAPA and IS in the plasma samples of the T2DM rats administered DAPA and ARE. DAPA, dapagliflozin; EMPA, Empagliflozin; IS, Internal standard.

Typical chromatograms of DAPA and IS in the blank plasma, the blank plasma spiked with the analytes (7.7 ng/mL) and IS (427.5 ng/mL), and plasma samples of the rats after oral administration of DAPA were presented in Figure 1. The retention times of DAPA and IS were 5.49 min and 4.22 min, respectively. There was no apparent interference from the endogenous substances at the retention times of the analytes and IS.

#### 3.2.2 Calibration curve, LOD and LLOQ

Calibration curve with the concentration ranges of 7.7–1950 ng/mL for DAPA was found to be linear regression. LOD of DAPA was determined as 2.15 ng/mL. LLOQ of DAPA was determined as 7.7 ng/mL. The typical calibration curve was *Y* = 0.000509 *X* + 0.0142 (*r* = 0.9972). Precision (*RSD*) and accuracy (*RE*) of the actual concentration at all the points on the standard curves were less than 15% of the standard concentration, including the LLOQ, which met the bioanalysis criteria.

#### 3.2.3 Precision and accuracy

The intra-day and inter-day precision and accuracy of the assayed method were summarized in Table 1. The intra-day and inter-day precision ranged from 3.04% to 7.73% and from 2.81% to 9.13%, respectively. The intra-day and inter-day accuracy fell in the ranges from 0.57% to 3.04% and -7.64%–2.05%, respectively. Precision and accuracy of the method met the requirements of validation.

**TABLE 1:** Inter-day and intra-day precision and accuracy of DAPA in the rat plasma (*n* = 6, mean ± SD).

Concentration of DAPA (ng/mL)	Intra-Day			Inter-Day		
	Measured (ng/mL)	Precision (*RSD*, %)	Accuracy (*RE*, %)	Measured (ng/mL)	Precision (*RSD*, %)	Accuracy (*RE*, %)
975	990.2±33.82	3.42	1.56	963±32.09	3.33	1.23
244	245.4±7.47	3.04	0.57	249.00±7.00	2.81	2.05
33.5	33.96±2.36	6.95	1.37	32.84±2.46	7.48	1.97
7.62	7.85±0.61	7.73	3.04	7.04±0.64	9.13	7.64

#### 3.2.4 Matrix effect and extraction recovery

Matrix effect and extraction recovery of the analyte were summarized in Table 2. Matrix effect ranged from 91.88% to 103.04%. *RSD* of the IS-normalized matrix effects did not exceed 9.13%, and influence of the biological matrix on the analyte’s response was negligent. Extraction recovery in the rat plasma samples at low and high concentration levels was greater than 94.07%.

**TABLE 2:** Matrix effects and extraction recovery of DAPA in the rat plasma (*n* = 6, mean ± SD).

Concentration of DAPA (ng/mL)	Extraction recovery		Matrix effect	
	Mean (%)	*RSD* (%)	Mean (%)	*RSD* (%)
975	98.04±3.35	3.42	103.04±3.13	3.04%
33.5	94.07±6.54	6.95	91.88±8.39	9.13%

#### 3.2.5 Stability

Stability of DAPA in the rat plasma under different storage and processed conditions were summarized in Table 3. DAPA was stable at the room temperature (25°C) for 12 h, 4°C for 24 h, -80°C for 30 days, and three cycles of freezing-thawing from -80°C to room temperature. *RE* and *RSD* values were within an acceptable range (±15%).

**TABLE 3:** Stability of DAPA in the rat plasma under different conditions (*n* = 6, mean ± SD).

	Concentration (ng/mL)	Accuracy (*RE*, %)	Precision (*RSD*, %)
Room temperature (25°C, 12 h)	975	-2.87	2.95
	33.5	-1.19	6.45
Refrigerator (4°C, 24 h)	975	-2.63	2.55
	33.5	-1.37	3.97
Freeze thaw (3 cycles)	975	-3.71	2.26
	33.5	-4.72	4.69
Long term (-80°C, 30d)	975	-2.97	1.92
	33.5	-2.96	4.30

Note, freeze thaw (3 cycles) referred to three freeze-thawing cycles, i.e., the samples were frozen for 7 d at -80°C and thawed 3 h at room temperature for each cycle.

### 3.3 Pharmacokinetics

#### 3.3.1 Pharmacokinetic differences of DAPA in healthy rats and T2DM rats

**FIGURE 2:**
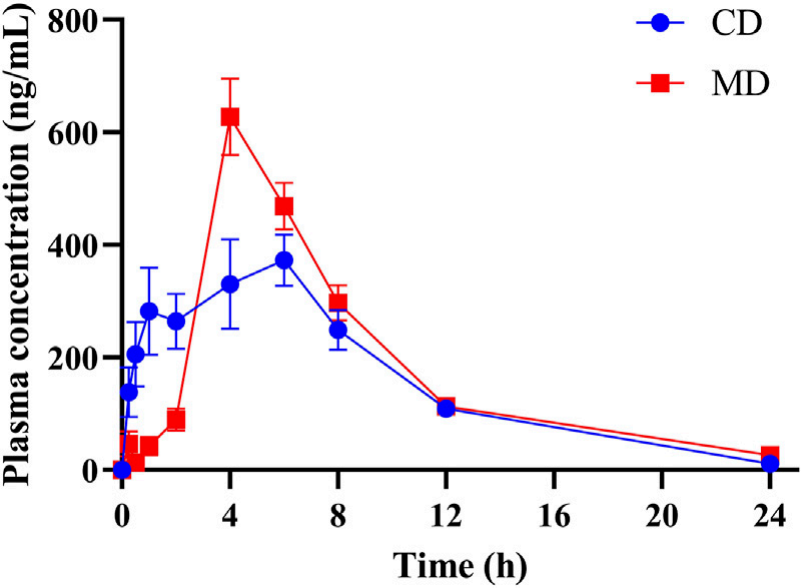
The mean plasma concentration-time profiles of DAPA in the healthy and T2DM rats, respectively (*n* = 6, mean ± SD). CD group, the healthy rats intragastrically administered with DAPA in a single dose (1.05 mg/kg). MD group, the T2DM rats intragastrically administered with DAPA in a single dose (1.05 mg/kg).

Mean plasma concentration-time profiles of DAPA in healthy and T2DM rats were shown in Figure 2, and the pharmacokinetic parameters of DAPA were summarized in Table 4. Compared to that in health status, T2DM status significantly increased *C*_max_ of DAPA by 62.94%, while *t*_max_ was significantly decreased by 29.45%. However, there were no significant changes for *t*_1/2_, *V*, AUC, CL and MRT in healthy rats and T2DM rats. This indicated that T2DM disease status significantly affected pharmacokinetic behavior of DAPA, i.e., promoted absorptive rate of DAPA, and increased its plasma concentrations.

**TABLE 4:** Pharmacokinetic parameters of DAPA alone or in combination with ARE in healthy and T2DM rats (*n* = 6, mean ± SD).

Parameter	CD	MD	CDA	MDA
*t*_1/2_ (h)	3.61±0.21	4.45±0.90	3.80±0.22	9.69±3.47^###^
*t*_max_ (h)	5.67±0.82	4.00±0.00^*^	2.50±1.22^****^	1.50±0.77^###^
*C*_max_ (ng/mL)	385.00±45.96	627.33±68.62^****^	504.33±99.44^*^	426.67±58.92^###^
*V* (mL/kg)	1425.95±163.28	1498.98±307.43	1570.40±401.41	3091.73±1183.58^##^
AUC_(0-t)_ (h*(ng/mL))	3810.20±343.81	4324.28±185.47	3783.24±852.77	4051.27±952.61
AUC_(0-inf)_ (h*(ng/mL))	3868.26±344.05	4505.74±148.96	3845.72±851.15	4865.13±1307.11
CL ((mL/h)/kg)	273.31±25.26	233.25±7.80	283.83±58.84	228.25±56.10
MRT_(0-t)_ (h)	6.89±0.28	7.57±0.32	5.16±0.36^****^	7.64±0.70

Note, CD group, the healthy rats intragastrically administered with DAPA in a single dose (1.05 mg/kg). CDA group, the healthy rats intragastrically administered with ARE (300 mg/kg) once a day for seven consecutive days, and intragastrically administered with DAPA in a single dose (1.05 mg/kg) at the same time on the seventh day. MD group, the T2DM rats intragastrically administered with DAPA in a single dose (1.05 mg/kg). MDA group, the T2DM rats intragastrically administered with ARE (300 mg/kg) once a day for seven consecutive days, and intragastrically administered with DAPA in a single dose (1.05 mg/kg) at the same time on the seventh day. ^*^ *p* < 0.05, ##*p* < 0.01, ###*p* < 0.001, ****p < 0.0001. * represent MD or CDA compared with CD; # represent MDA compared with MD.

#### 3.3.2 Pharmacokinetic differences of DAPA used alone or in combination with ARE

**FIGURE 3:**
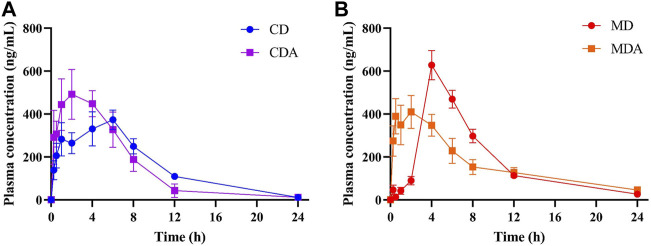
The mean plasma concentration-time profiles of DAPA alone or in combination with ARE in the healthy and T2DM rats, respectively (*n* = 6, mean ± SD). **(A)**, DAPA was administered alone or in combination with ARE for 7 days in the healthy rats. **(B)**, DAPA was administered alone or in combination with ARE for 7 days in T2DM rats. CD group, the healthy rats intragastrically administered with DAPA in a single dose (1.05 mg/kg). CDA group, the healthy rats intragastrically administered with ARE (300 mg/kg) once a day for seven consecutive days, and intragastrically administered with DAPA in a single dose (1.05 mg/kg) at the same time on the seventh day. MD group, the T2DM rats intragastrically administered with DAPA in a single dose (1.05 mg/kg). MDA group, the T2DM rats intragastrically administered with ARE (300 mg/kg) once a day for seven consecutive days, and intragastrically administered with DAPA in a single dose (1.05 mg/kg) at the same time on the seventh day.

**FIGURE 4:**
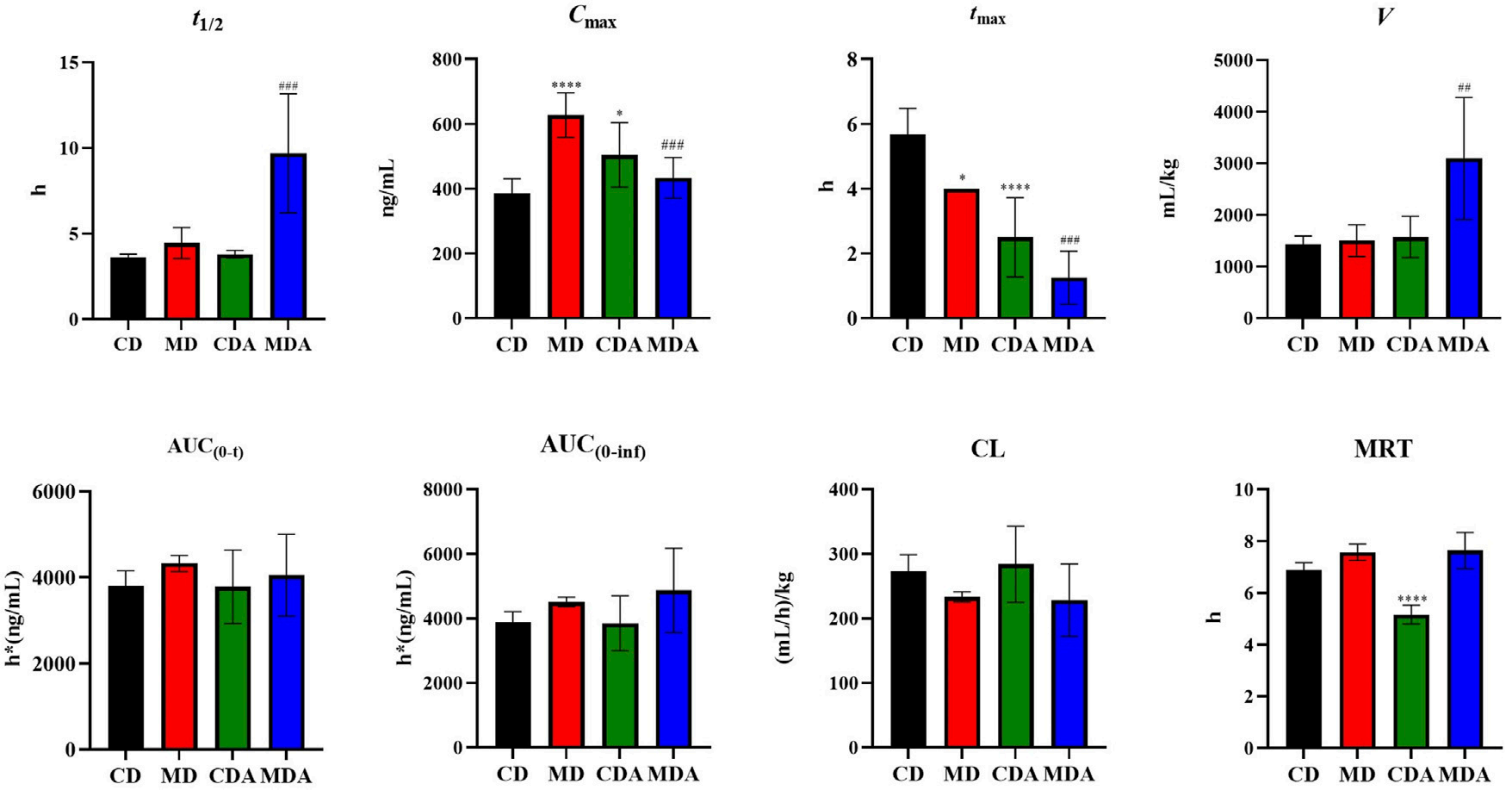
Pharmacokinetic parameters of DAPA alone or in combination with ARE in the healthy and T2DM rats (*n* = 6, mean ± SD). CD group, the healthy rats intragastrically administered with DAPA in a single dose (1.05 mg/kg). CDA group, the healthy rats intragastrically administered with ARE (300 mg/kg) once a day for seven consecutive days, and intragastrically administered with DAPA in a single dose (1.05 mg/kg) at the same time on the seventh day. MD group, the T2DM rats intragastrically administered with DAPA in a single dose (1.05 mg/kg). MDA group, the T2DM rats intragastrically administered with ARE (300 mg/kg) once a day for seven consecutive days, and intragastrically administered with DAPA in a single dose (1.05 mg/kg) at the same time on the seventh day. ^*^ *p*, ^#^ *p* < 0.05, ^**^ *p,* ^##^ *p* < 0.01, ^***^ *p*, ^###^ *p* < 0.001, ^****^ *p*, ^####^ *p* < 0.0001. * represent MD or CDA compared with CD, ^#^ represent MDA compared with MD.

Mean plasma concentration-time profiles of DAPA after oral administration alone and in combination with ARE in healthy and T2DM rats were shown in Figure 3. Main pharmacokinetic parameters of DAPA were displayed in Table 4. Compared to that in the CD group, *t*_max_ and MRT of DAPA decreased by 55.91% and 25.11%, respectively, and *C*_max_ of DAPA increased by 30.99% in the CDA group. However, *t*_1/2_, AUC_(0-t)_, AUC_(0-inf)_, *V* and CL showed no significant difference between the CD group and the CDA group (Figure 4). This indicated that ARE shortened *t*_max_ and MRT, increased *C*_max_ of DAPA in the healthy rats when they were co-administrated, but did not affect the other pharmacokinetic parameters of DAPA.

Compared to that in the MD group, *t*_max_ and *C*_max_ of DAPA decreased by 62.50% and 31.99% respectively, *t*_1/2_ and *V* of DAPA were increased by 2.18- and 2.06-fold respectively in the MDA group. However, AUC_(0-t)_, AUC_(0-inf)_, CL, MRT showed no significant difference between the MD group and the MDA group (Figure 4). This suggested that ARE can short *t*_max_ and *C*_max_ of DAPA, extended its *t*_1/2_, promoted its distribution to target tissues and organs in the T2DM rats. However, it did not affect the other pharmacokinetic parameters of DAPA.

## 4 Discussion

Healthy animals or volunteers are usually selected as the subjects for preclinical and phase 1 clinical trials of drugs. However, drugs are usually applied to the patients. Therefore, the pharmacokinetic experimental results of animals or humans in healthy status sometimes cannot truly reflect the pharmacokinetic process of drugs in diseased status. Many studies have shown that some diseases (e.g., liver disease, kidney disease, digestive tract disease, DM (Galicia-Garcia et al., 2020)) can change physiological and pathological state of the body, and further affect pharmacokinetic processes of drugs in the body.

As a long term, chronic metabolic disease, DM can damage multiple tissues and organs in the body, e.g., kidney, liver, gastrointestinal tract and blood vessels, thus, affect absorption, distribution, metabolism and excretion of some drugs (e.g., antidiabetic drugs), further affect efficacy and safety of drugs. As a new antidiabetic drug, DAPA is more and more used in clinical practice. Pharmacokinetics of DAPA have been studied in some aspects, e.g., effect of age, race, gender, body weight, mild renal or hepatic impairment on pharmacokinetics of DAPA. However, up to now, effect of disease status of DM on pharmacokinetic behavior of DAPA has not been investigated, pharmacokinetic difference of DAPA in healthy and T2DM status has also not been systematically elucidated. So, pharmacokinetic differences of DAPA in healthy and T2DM rats were studied and compared so as to disclose effect of T2DM on pharmacokinetic behavior of DAPA *in vivo* in this study.

Our study indicated that, pharmacokinetic behavior of DAPA showed significant difference in health and T2DM status. Compared to that in the healthy rats, *C*_max_ of DAPA significantly increased, and its *t*_max_ significantly decreased in the T2DM rats. This means that disease status of T2DM increased maximum plasma drug concentration of DAPA *in vivo* and shortened time to achieve *C*_max_ and maximum effect. It was reported that, expression of P-glycoprotein (P-gp) can be inhibited in the disease status of T2DM (Zhang et al., 2011). DAPA is substrate of P-gp (Obermeier et al., 2010). Therefore, it may be deduced that, P-gp-mediated intestinal efflux of DAPA can be reduced in the status of T2DM by inhibiting expression of P-gp. Further, plasma concentration and absorptive rate of DAPA *in vivo* was increased, which was conducive to enhance hypoglycemic effect of DAPA. This was consistent with our experimental results.

Damage of liver and kidney often affects metabolism and excretion of drugs, further affects clearance of drugs, finally affects efficacy and safety of drugs. Following with progression of T2DM, it often affects functions of liver and kidney, lead to hepatic and renal dysfunction. Hepatic and renal dysfunction induced by T2DM further affects pharmacokinetic behavior of drug (e.g., DAPA) *in vivo*. Both liver and kidney involve in metabolic clearance of DAPA, and form the predominant metabolite DAPA 3-*O*-glucuronide. Similarly, severe hepatic or renal impairment affect plasma drug concentration of DAPA *in vivo*. It had been confirmed that *C*_max_ of DAPA were significantly increased in T2DM patients with liver and kidney dysfunction (Kasichayanula et al., 2014). Compared to that in healthy subjects without liver function damage, *C*_max_ of DAPA increased 12%, 12%, and 40% in the patients with mild, moderate and severe liver function damage, respectively (Kasichayanula et al., 2011). Compared to that in T2DM patient without renal function damage, *C*_max_ of DAPA increased 4%, 6% and 9% in T2DM patients with mild, moderate and severe renal function damage, respectively (Kasichayanula et al., 2013). Our study showed that, compared to that in healthy rats, *C*_max_ of DAPA increased 62.94% in T2DM rats. This implied that T2DM rats induced by HFD combined with STZ may cause hepatic and renal impairment of the rats, further affected pharmacokinetic behavior of DAPA. Therefore, T2DM patients with liver or kidney impairment need to adjust dosage of DAPA according to the measured pharmacokinetic parameters so as to avoid some potential risks of adverse events (e.g., hypoglycemia).

Co-administration of antidiabetic drugs (e.g., biguanides, sulfonylureas, glinides) and herbs/dietary supplement (e.g., AR, *Rehmannia glutinosa*, *Pueraria lobata* and their compound preparation) is very popular in clinical practice in some countries (e.g., China, Japan) (Cui et al., 2018; Wu et al., 2021; Liu et al., 2023). DAPA is frequently co-administrated with some popular antidiabetic herbs (e.g., AR and its compound preparation) in China. *Astragali Radix* or *Astragali Radix*-containing formulas are widely used to treat T2DM or diabetic nephropathy in Asia which display favorable efficacy and safety (Li et al., 2011; Chan et al., 2021). At present, there is no study of pharmacokinetic interaction of DAPA and herbs, especially effect of AR on pharmacokinetics behavior of DAPA *in vivo*. Therefore, the related studied were conducted using healthy and T2DM rats in the present study, respectively.

This study found that ARE shortened *t*_max_ and MRT, and increased *C*_max_ of DAPA in healthy rats when ARE and DAPA were co-administered, but did not affect eliminate, distribution and exposure of DAPA *in vivo*. Changes of pharmacokinetic parameters of DAPA may be related to effect of AR components on P-gp-mediated DAPA efflux. As one of the main active constituents of AR, *Astragalus* polysaccharides may decrease P-gp-mediated efflux of DAPA in dose-dependent manner by inhibiting express of P-gp (Tian et al., 2012). Astragaloside II derived from AR and ARE is another potent P-gp inhibitor, and can be used a potential adjunctive agent of antidiabetic drugs (Huang et al., 2012). Thus, some active components in ARE promoted absorption of DAPA by inhibiting P-gp, and changed the pharmacokinetic behavior of DAPA. Moreover, it was reported that, after single oral administration of ARE, its main constituents (i.e., calycosin-7-β-glucoside, formononetin, ononin, and astragaloside Ⅳ) and glucuronide metabolites (i.e., calycosin-3′-glucuronide, calycosin-7-β-glucoside-3′-glucuronide, formononetin-7-glucuronide, daidzein-7-glucuronide) were exposed in the rat plasma (Shi et al., 2015). These constituents and their glucuronide metabolites possibly play important role in changing pharmacokinetic behavior of DAPA by regulating transporters (e.g., SGLT2, P-gp, MRP2, BCRP), metabolic enzymes (e.g., CYP1A1, CYP1A2, CYP2A6, CYP2C9, CYP2D6, CYP3A4, UGT1A9, UGT2B4 and UGT2B7) and gut microbiota (Obermeier et al., 2010; Kasichayanula et al., 2014) when ARE was co-administrated with DAPA.

Similarly, ARE promoted intestinal absorption of DAPA and shortened its *t*_max_ in T2DM rats. In addition, ARE significantly increased *V* and *t*_1/2_ of DAPA, significantly decreased *C*_max_ of DAPA, but unchanged AUC of DAPA in T2DM status. It should be noted that, ARE changed some pharmacokinetic characteristics (e.g., plasma drug concentration, *t*_max_, C_max_) of DAPA in healthy and T2DM rats, but it did not show obvious dose-dependence since there was no significant change of pharmacokinetic parameters of DAPA with increase of ARE dose from 300 mg/kg to 900 mg/kg (Supplementary Figure S1 and Supplementary Table S1).

A number of clinical studies have shown that AR can improve kidney function, and reduce proteinuria (Tang and Zhang, 2022). Therefore, protective effect of AR on renal function may reduce the elevated *C*_max_ of DAPA which is caused by renal impairment. Mean *V* of DAPA combined with ARE was 2915.86 mL/kg in T2DM rats, which was much greater than the estimated plasma volume for the rats. It means that ARE can promote extravascular tissue distribution of DAPA. ARE increased *t*_1/2_ of DAPA by 2.18 times, and prolonged action time of DAPA, but did not lead to accumulation of DAPA since AUC _(0-t)_ or AUC _(0-inf)_ of DAPA remained unchanged. Therefore, co-administration of ARE and DAPA does not cause undesirable pharmacokinetic interactions, and may promote its antidiabetic efficacy.

Anyway, T2DM is a complex condition with a variety of causes and pathophysiology. The current single target approach has not provided ideal clinical outcomes for the treatment of the disease and its complications. The global use of herbs for the management of T2DM has rapidly increased over the last decade. Herbs exert favorable antidiabetic effect based on their multi-components, multi-targets and multi signal pathways. Co-administration of antidiabetic herbs (e.g., ARE) and drugs (e.g., DAPA) can realize complementary advantages and present therapeutic benefit by alter their pharmacokinetic and/or pharmacodynamic properties. It is undeniable that, their co-administration sometimes also increases the potential adverse event risks (e.g., potential harmful herb-drug interactions) (Gupta et al., 2017). On the whole, after co-administration of ARE and DAPA, the potential drug-drug interactions between ARE and DAPA can improve their antidiabetic effect and reduce risk of adverse reactions, which will provide beneficial guidance for their clinical application.

In addition, it needs to noted that, it is crucial to establish an accurate, sensitive, stable and reliable biological sample analysis method for *in vivo* pharmacokinetic study. A simple, sensitive and reliable LC-MS/MS method for the determination of DAPA and empagliflozin in the rat plasma was developed and validated in this study. Matrix effect and extraction recovery have significant impact on the accuracy and reliability of the assayed results. During the sample preparation process, matrix effects can affect stability of drugs, increase LOD of drugs, and reduce sensitivity of drugs. Compared to the protein precipitation with methanol and acetonitrile, liquid-liquid extraction with MTBE minimized the interference of endogenous substances in plasma samples and contributed to enrich the analytes. As a favorable extractant, MTBE was used to extract DAPA in the rat samples and obtained excellent recovery (≥94.07%). 1 mM ammonium formate was added to the mobile phase to allow DAPA to form ammoniated adduct ions [M + NH4]^+^ in the positive mode with stronger signal compared with protonated ions [M + H]^+^ in the present study. 0.1% formic acid was added into the mobile phase which increased sharp and symmetrical peak of DAPA.

This study explored effects of disease status of T2DM and co-administration of herbs (i.e., ARE) on pharmacokinetic behavior of DAPA. However, some limitations should be noted that, this study did not examine effects of DAPA on pharmacokinetic behavior of ARE and its components. Additionally, underlying interaction mechanisms of ARE and DAPA, e.g., the above-mentioned transporter and metabolic enzyme-mediated interaction of DAPA and ARE as well as its components, have not been validated by further rigorous scientific research (e.g., cellular, molecular biology and animal experiments) in the present study.

## 5 Conclusion

In summary, T2DM status and co-administration with ARE significantly affected pharmacokinetic behaviors of DAPA *in vivo*. If necessary, the patients with T2DM as well as its complications should adjust the administered dose when ARE and DAPA are co-administered. Co-administration of antidiabetic herbs and drugs should be recommended when their co-administration displayed favorable synergistic or additive antidiabetic effect and no or acceptable risk of adverse events.

## Data Availability

The original contributions presented in the study are included in the article/Supplementary Material, further inquiries can be directed to the corresponding author.

## Abbreviations

DM, diabetes mellitus; T1DM, type 1 diabetes mellitus; T2DM, type 2 diabetes mellitus; DPP-4, dipeptidyl-peptidase 4; GLP-1, glucagon-like peptide-1; SGLT-2, sodium-dependent glucose transporters 2; DAPA, dapagliflozin; AR, Astragali Radix; ARE, Astragali Radix extract; IS, internal standard; QC, quality control; LQC, low quality control; MQC, medium quality control; HQC, high quality control; STZ, streptozotocin; MTBE, methyl tert-butyl ether; LOD, limit of detection; LLOQ, lower limit of quantification; HFD, high-fat diet; FBG, fasting blood glucose; t1/2, time required to eliminate half of plasma drug concentration; tmax, time to maximum plasma concentration; Cmax, maximum plasma concentration; AUC, area under the concentration–time curve; V, apparent volume of distribution; CL, clearance of drug plasma volume per time unit; MRT, mean residence time; P-gp, P-glycoprotein; MRP2, multidrug resistant protein 2; BCRP, breast cancer resistance protein.
