# Supplementary material for: Effect of Astragali radix extract on pharmacokinetic behavior of dapagliflozin in healthy and type 2 diabetic rats
Source: Front Pharmacol. 2023 Oct 10;14:1214658. doi: 10.3389/fphar.2023.1214658 (PMC10597649; doi:10.3389/fphar.2023.1214658)
Supplement: Supplementary file 1 [file DataSheet1.docx]

**Supplementary Figure and Table**

**Supplementary Figure 1** The mean plasma concentration-time profiles of DAPA (*n*=6, mean ± SD) in combination with ARE. CDA-L, CDA-H group, the healthy rats were intragastrically administered with 300 mg/kg and 900 mg/kg of ARE once a day for seven consecutive days, respectively, and both of them were intragastrically administered with DAPA in a single dose (1.05 mg/kg) on the seventh day. MDA-L, MDA-H group, the T2DM rats were intragastrically administered with 300 mg/kg and 900 mg/kg of ARE once a day for seven consecutive days, respectively, and both of them were intragastrically administered with DAPA in a single dose (1.05 mg/kg) on the seventh day.

**Supplementary Figure 1**


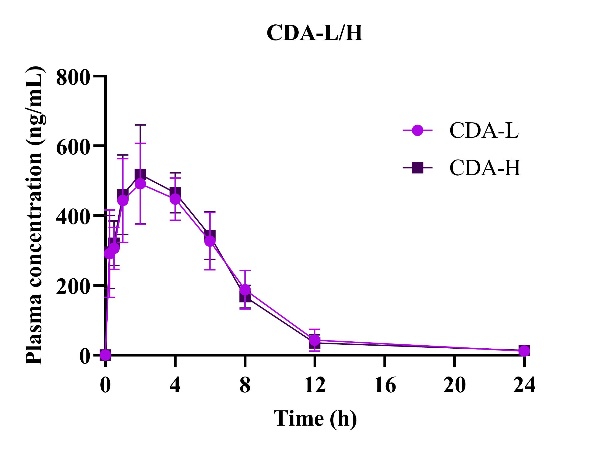

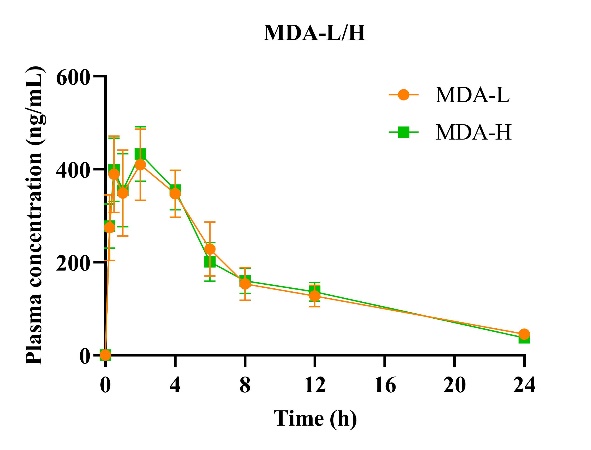


**Supplementary Table 1** Pharmacokinetic parameters of DAPA in combination with 300 mg/kg and 900 mg/kg of ARE in healthy and T2DM rats, respectively (*n*=6, mean ± SD).

| Parameter | CDA-L | CDA-H | MDA-L | MDA-H |
| --- | --- | --- | --- | --- |
| *t*_1/2_ (h) | 3.80±0.22 | 3.96±0.17 | 8.64±1.08 | 7.44±0.86 |
| *t*_max_ (h) | 2.50±1.22 | 2.61±0.90 | 1.25±0.82 | 1.45±0.61 |
| *C*_max_ (ng/mL) | 504.33±99.44 | 538.00±122.61 | 433.33±62.61 | 456.83±50.42 |
| AUC_(0-t)_(h*(ng/mL)) | 3783.24±852.77 | 3868.01±745.36 | 3991.37±640.65 | 4070.47±408.83 |
| AUC_(0-inf)_(h*(ng/mL)) | 3845.72±851.15 | 3946.77±756.89 | 4560.99±768.53 | 4464.67±408.26 |
| V (mL/kg) | 1570.40±401.41 | 1554.45±219.69 | 2915.86±510.24 | 2552.48±482.45 |
| CL ((mL/h)/kg) | 283.83±58.84 | 273.49±46.98 | 235.15±35.35 | 237.88±21.93 |
| MRT_(0-t)_ (h) | 5.16±0.36 | 5.04±0.27 | 7.39±0.34 | 7.23±0.37 |

Note, CDA-L, CDA-H group, the healthy rats were intragastrically administered with 300 mg/kg and 900 mg/kg of ARE once a day for seven consecutive days, respectively, and both of them were intragastrically administered with DAPA in a single dose (1.05 mg/kg) on the seventh day. MDA-L, MDA-H group, the T2DM rats were intragastrically administered with 300 mg/kg and 900 mg/kg of ARE once a day for seven consecutive days, respectively, and both of them were intragastrically administered with DAPA in a single dose (1.05 mg/kg) on the seventh day.
